# City-level synergy and co-benefits of mitigating CO_2_ emissions and air pollution in China

**DOI:** 10.1016/j.heliyon.2024.e34667

**Published:** 2024-07-17

**Authors:** Li Zhang, Linyi Wei, Jiaqi Ren, Zhe Zhang, Ruxing Wan, Shuying Zhu, Bofeng Cai, Jinnan Wang

**Affiliations:** a Ministry of Education Key Laboratory for Earth System Modeling, Department of Earth System Science, Tsinghua University, Beijing, 100084, China; b School of Environment, Beijing Normal University, Beijing, 100875, China; c Center for Carbon Neutrality, Chinese Academy of Environmental Planning, Beijing, 100043, China; d School of Economics and Management, Beijing University of Chemical Technology, Beijing, 100029, China

**Keywords:** Index, Synergistic control, Economic co-benefits, Chinese city, CO_2_, PM_2.5_

## Abstract

Synergistic control of carbon emissions and pollutant concentrations can efficiently tackle climate change and air pollution. The synergistic performance and co-benefits yielded by controlling these factors are urgent and critical issues in China. Accordingly, a comprehensive indicator for assessing synergistic performance is pioneered, and co-benefits of mitigating CO_2_ and PM_2.5_ in Chinese cities are evaluated. Chinese synergistic performance is dominated by CO_2_ variations. In 2015–2020, multiple northeastern, central, southern, and eastern cities exhibited synergistic effects by greatly reducing CO_2_ emissions. The synergistic performance thereafter leads to co-benefits through environmental and economic feedbacks. The CO_2_ and PM_2.5_ controls in Northeast Chinese cities feature the most profound co-benefits of approximate 4800 CNY per capita, with each contributing 72 % and 28 %, respectively, to this total. The findings provide intercity synergistic performance and scientific support for policymaking.

## 1 Introduction

Mitigating climate change and improving air quality are two important challenges that China is facing. As the largest carbon emitter in the world [1], the energy structure of China is still dominated by fossil fuels, which has triggered the consistent growth of carbon dioxide (CO_2_) emissions in recent years [2]. This increase in CO_2_ and the accompanying greenhouse effect have further exacerbated climate change. In tackling climate change, China has committed to reaching peak CO_2_ emissions before 2030 and achieving carbon neutrality by 2060. On the other hand, China is also under pressure to improve its air quality. Although China has made great efforts and significant improvements in its air quality since 2013 [3], in 2021, approximately 36 % of Chinese cities exceeded the National Ambient Air Quality Standards for PM_2.5_ (particulate matter with an aerodynamic diameter ≤2.5μm) (i.e., 35 μg/m^3^) [4], let alone the World Health Organization standard of 5 μg/m^3^ [5].

With the context of reducing greenhouse gas (GHG) emissions and pollution, promoting their synergistic control has drawn more attention from both researchers and policymakers. In the past, climate change mitigation and air quality improvement were treated as two separate issues in China [6]. However, substantial evidence has indicated that GHGs (e.g., CO_2_) and air pollutants (e.g., PM_2.5_), especially from the same sources, could be controlled synergistically to meet comprehensive targets on emission reductions [7,8]. To promote synergistic control of GHGs and air pollution, China issued a top-level policy in 2022, namely, the Implementation Plan for the Synergistic Effect of Pollution Reduction and Carbon Reduction [9], and highlighted its importance at the 20th National Congress [10].

Harmonized evaluation criteria are essential for governments to make synergistic emission reduction decisions. National and provincial governments need to develop future emission reduction policies in a unified manner, and local environmental managers need a clear understanding of the emission status of their cities compared to other cities. Therefore, a custom indicator, evaluating the synergistic effect of reducing air pollutants and CO_2_ emissions, is necessary. Furthermore, the performance of synergistic control and the benchmark for future policymaking has been generally overlooked in previous studies.

Evaluation indicators of CO_2_ and air pollution controls have increased in recent years. Some indicators only focused on a certain environmental index, either CO_2_ or PM_2.5_. For example, some scholars have used carbon intensities [[11], [12], [13]] and per capita CO_2_ emissions [14] to measure regional low-carbon development levels, and the concentration (μg/m^3^) is commonly used to evaluate PM_2.5_ emissions [15]. These measures ignore the synergistic control effect of CO_2_ mitigation and air pollution improvement. Other indicators consider multiple factors from economic, social, and environmental perspectives [16,17]. Comprehensive yet complex index systems have been adopted to analyze the synergies and trade-offs of the economic‒social‒environment system [18]. For example, Qiao et al. established a co-benefit indicator system including 23 indicators to evaluate the co-benefits of city policies with regard to energy conservation and environmental sustainability based on the driver‒pressure‒state‒impact‒response framework [19]. Indicator systems usually cover different dimensions and various factors, relying on data availability. However, a less expensive and time-saving approach for constructing a unified synergy control index is still lacking.

Moreover, existing evaluation indicators have focused mainly on the synergy between CO_2_ mitigation and air pollution control at the national [20], provincial [16], and regional [21] level but rarely at the city level [22]. Cities in China, which are the fundamental administrative units for implementing control policies [23], exhibit great heterogeneity in terms of economic, social, and environmental aspects [24] and provide unique contribution to national goals and policies. However, only a few evaluation indicators can accurately assess the degree of city synergistic control due to the fundamental data being unavailable and less quality-controlled [25].

By synergy between energy conservation and pollution reduction (mitigating GHG emissions and reducing pollution emissions), co-benefits in terms of the environment, economy, and health can be driven [10]. A growing number of studies have investigated the co-benefits from controlling CO_2_ emission-related social costs and PM_2.5_-related health impacts [26]. Some studies have shown that air pollution can benefit from CO_2_ emission reduction, thus peaking CO_2_ before 2030 could yield considerable co-benefits of health by reducing PM_2.5_ levels and related mortality [15,20,27]. Conversely, limiting the spread of SO_2_, soot, dust, and other primary pollutants has synergistic impacts on the control of GHG emissions [28,29]. Decoupling analysis can be used to analyze the relationship between environmental pressure and economic growth [24,30]. According to the calculated decoupling elastic values, we divide cities into different regions and identify the factors influencing the synergistic control. In addition, we discuss the economic feedback of synergistic control, which is crucial for the future development of cities.

PM_2.5_ and CO_2_ can impact the social economy by threatening public health and climate change. PM_2.5_ causes economic co-losses by increasing health risks and premature deaths [31]. On the one hand, premature mortality reduces the investment in protecting health, as widely measured by the value of statistical life (VSL, the marginal cost to reduce the risk of death) [[32], [33], [34]]. On the other hand, the labor force due to premature deaths decreases productivity, quantified by multiplying dead labor and marginal productivity [[35], [36], [37]]. The economic impact of CO_2_ is related to the carbon price and is usually quantified in terms of the social cost of carbon (SCC), which characterizes the marginal cost per unit of CO_2_ emissions needed to mitigate climate change [[38], [39], [40]]. The SCC dramatically varies from $10 to $1000 per ton of CO_2_ according to different approaches and models [38,41], with the Intergovernmental Panel on Climate Change (IPCC) recommended value of approximately $100 (/tCO_2_) being the most widely used [42].

Exploring the factors influencing synergistic control is crucial for reducing pollution and carbon emissions in cities. Some scholars have discussed the influences of specific engineering technologies or policies on economic activities and associated emissions of GHGs and air pollutants [43,44]. The logarithmic mean Divisia index (LMDI) method is often used to explore the factors driving emission reductions [30,45]. In addition, there might be spatial correlations in PM_2.5_ and CO_2_ between adjacent cities. Spatial econometric models can consider spatial correlations more effectively than conventional regression models such as the ordinary least squares (OLS) model [46]. To determine the factors influencing synergistic control and to assist cities in achieving synergistic emission reduction, we explore the forces driving changes in CO_2_ emissions and PM_2.5_ concentrations using the LMDI and spatial econometric models.

The novelty of this study includes the following three aspects. First, we innovatively construct a comprehensive synergistic control index to assess the city-level synergistic performance of CO_2_ mitigation and air pollution control. Based on this index, we cover almost all prefecture-level cities in China, quantify the synergy degrees of all cities from 2015 to 2020, and define the theoretical and practical implications of the index. Second, we evaluate economic feedback to synergistic controls and integrate the synergistic results from an economic benefit perspective. Third, we select representative cities with various development levels, explore the influencing factors and mechanisms of city-level synergies, and help cities formulate synergy emission reduction policies under different basic conditions. Our research provides a new benchmark for cities to reduce air pollution and carbon emissions synergistically, thereby supporting the sustainable development of cities and meeting national climate and clean air goals.

## 2 Materials and methods

### 2.1 Ranking of city-level synergetic emission reductions

The average ranking method was used to evaluate the degree of synergetic reduction in pollution and carbon emissions. The average ranking method could directly transform a multi-index evaluation method into a single-index evaluation method to eliminate the influences of extreme values and describe the relative position rather than the quantity of the evaluation factors. In this study, PM_2.5_ concentrations and CO_2_ emissions, as two paramount environmental metrics, are utilized to represent air pollution and carbon emission levels, respectively. As an indicator of changes in pollutants and carbon emissions, the relative rate of change in the average annual PM_2.5_ concentrations and CO_2_ emissions from 2015 to 2020 were selected as the basis for ranking. The 335 cities were ranked by the following steps. 1) The relative change rates of PM_2.5_ concentrations and CO_2_ emissions from 2015 to 2020 were calculated by using Equations (1), (2). 2) The 335 cities were ranked (in the ascending order) based on the results, and the average values of the rankings were taken as the final values in Equation (3).

(1) $$\Delta_{r}{\text{PM}}_{2.5}=\frac{{\text{PM}}_{2.5,2020}-{\text{PM}}_{2.5,2015}}{{\text{PM}}_{2.5,2015}}$$

(2) $$\Delta_{r}{\text{CO}}_{2}=\frac{{\text{CO}}_{2,2020}-{\text{CO}}_{2,2015}}{{\text{CO}}_{2,2015}}$$

where Δr PM_2.5_ is the relative rate of change in PM_2.5_; PM_2.5,2020_ and PM_2.5,2015_ are the annual average concentrations of PM_2.5_ (μg/m^3^) in 2020 and 2015, respectively; Δr CO_2_ is the relative rate of change in CO_2_; and CO_2,2020_ and CO_2,2015_ are the CO_2_ emissions (10^4^ tons) in 2020 and 2015, respectively.

(3) $$R=\frac{R_{{CO}_{2}}+R_{{PM}_{2.5}}}{2}$$

where R is the final ranking of cities, $R_{{CO}_{2}}$ is the ranking of $\Delta_{r}{CO}_{2}$ from small to large in the ascending order from 1 to 335, and $R_{{PM}_{2.5}}$ is the ranking of $\Delta_{r}{PM}_{2.5}$ from small to large in the ascending order from 1 to 335.

### 2.2 Benefits of pollution control and CO_2_ emission mitigation

Exposure to high PM_2.5_ concentrations could significantly impact the respiratory system and lung passageways, leading to a variety of health issues and health-economic losses [47]. Based on the policy goal of decreasing carbon emissions, the increase in CO_2_ emissions could imply potential costs. In contrast, co-benefits can be yielded by mitigating PM_2.5_ concentrations and carbon emissions. We estimated the co-benefits of pollution control and CO_2_ emission mitigation in a monetized manner to intuitively quantify the influences of PM_2.5_ and CO_2_ reductions.

To assess the health impacts attributed to PM_2.5_, we applied the Global Exposure Mortality Model, which is widely used to evaluate the relationships between PM_2.5_ concentrations and nonaccidental deaths. The premature mortalities of four diseases caused by PM_2.5_, namely, chronic obstructive pulmonary disease (COPD), ischemic heart disease (IHD), lung cancer (LC), and stroke, were considered in this study. The health risks and the premature mortality (MOR) due to exposure to PM_2.5_ were calculated by Equations (4), (5):

(4) $${HR}_{i}=e^{\frac{\beta_{i}\;\log\;\left(\frac{z}{\alpha_{i}}+1\right)}{1+e^{-\left(z-\mu_{i}\right)}/\nu_{i}}}$$

(5) $${\text{MOR}}_{i}=B_{i}\times \text{POP}\times \left(\frac{{\text{HR}}_{i}-1}{{\text{HR}}_{i}}\right)$$

where i is the disease of COPD, IHD, LC or stroke; HR_i_ is the hazard ratio of premature mortality attributable to PM_2.5_ pollution of the specific disease i, z = max (0, PM_2.5_–2.4 μg/m^3^), indicating that 2.4 μg/m^3^ is the minimum concentration posing health risks, as recommended by Burnett et al. [48]; MOR_i_ is the mortality attributable to PM_2.5_ pollution (persons) of the specific disease i; B_i_ is the baseline mortality rate of disease i; POP is the permanent population; β is the exposure‒response model coefficient; and α, μ, and ν are parameters that control the curve of the PM_2.5_-related mortality. The values of parameters β, α, μ, ν, and B are shown in Table 1 from Burnett R et al. [48]. The PM_2.5_ and POP datasets used in this study are detailed in Section 2.5. B_i_ is publicly available from the Global Burden of Disease Study (GBD) (per 100,000 people) [49].

**Table 1:** Values of parameters in the Global Exposure Mortality Model.

Disease	β	α	μ	ν	B
COPD	0.251	6.5	2.5	32	80
LC	0.2942	6.2	9.3	29.8	40
IHD	0.2969	1.9	12	40.2	116
Stroke	0.272	6.2	16.7	23.7	80

To quantify the monetary value of the health benefits of PM_2.5_ reduction, a VSL of 5.24 million Chinese yuan (CNY) per person was used [50]. Thus, the product of VSL multiplied by MOR was the monetized health effect associated with PM_2.5_ exposure, indicating that air quality improvements induced co-benefits because of decreased health risks.

To measure the benefits associated with CO_2_ emission reduction, we adopted a social cost value of $185 per ton of CO_2_ [51] with a discount rate of 3 % [52] and applied an exchange rate of U.S. dollars to CNY at 7.

### 2.3 Decoupling analysis

Decoupling was used to describe the relationship between economic growth and environmental pressure. According to the latest report of the IPCC [53], the emission/economy decoupling method was proposed to evaluate the decoupling status of each city. The decoupling index (DI) was calculated based on changes in the GDP and CO_2_ emissions or the GDP and PM_2.5_ concentrations, as shown in Equation (6). DI = 1 was considered the turning point of absolute decoupling and relative decoupling, and the greater the DI value was, the lower the dependence of CO_2_ emissions or PM_2.5_ concentrations on economic growth. Absolute decoupling (DI > 1) referred to the decrease in CO_2_ emissions or PM_2.5_ concentrations as GDP increased. Relative decoupling (0 < DI < 1) suggested that the growth increase rates of CO_2_ emissions or PM_2.5_ concentrations were lower than that of the GDP. Non-decoupling (DI < 0) indicated that CO_2_ emissions or PM_2.5_ concentrations increased as fast or faster than the GDP.

(6) $$DI=\frac{△G\%-△E\%}{△G\%}=\left(\frac{G_{2020}-G_{2015}}{G_{2015}}-\frac{E_{2020}-E_{2015}}{E_{2015}}\right)/\frac{G_{2020}-G_{2015}}{G_{2015}}$$

where DI represents the decoupling index; G_2020_ represents the GDP in 2020; G_2015_ represents the GDP in 2015; E_2020_ represents the CO_2_ emissions or PM_2.5_ concentrations in 2020; and E_2015_ represents the CO_2_ emissions or PM_2.5_ concentrations in 2015.

### 2.4 Logarithmic mean divisia index decomposition analysis

To understand the driving forces of CO_2_ emission changes, we used LMDI, which decomposed CO_2_ emissions into the factors population, gross domestic product (GDP) per capita, industry structure, energy intensity, and energy structure as follows in Equation (7) [45]:

(7) $$C=\sum_{k=1}^{m}\sum_{j=1}^{n}POP\times \frac{GDP}{POP}\times \frac{{GDP}_{k}}{GDP}\times \frac{E_{k}}{{GDP}_{k}}\times \frac{E_{k,j}}{E_{k}}\times \frac{C_{k,j}}{E_{k,j}}$$

where C represents the CO_2_ emission, POP represents the city-level Permanent population, GDP represents the city-level or industry-level GDP, E represents the energy consumption, k represents industry or sector, and j represents energy type.

### 2.5 Spatial autocorrelation

By using the changes in CO_2_ emissions or PM_2.5_ concentrations at the city level, we further evaluated their spatial variations by global spatial autocorrelation and local spatial autocorrelation. First, spatial autocorrelation at the global level was conducted to investigate the characteristics of spatial association and heterogeneity [54]. The Global Moran's I is a comprehensive evaluation method for measuring spatial autocorrelation [55]. The significance of the Global Moran's I method could be tested using Z and p values. When |Z| ≤ 1.96 and p ≥ 0.05, I was considered not significant, when |Z| ≥ 1.96 and p < 0.05, I was considered significant. The Global Moran's I was calculated by Equations (8), (9):

(8) $$I=\frac{n\sum_{i=1}^{n}\sum_{j=1}^{n}W_{ij}\left(x_{i}-\bar{x}\right)\left(x_{j}-\bar{x}\right)}{\sum_{i=1}^{n}\sum_{j=1}^{n}W_{ij}\sum_{i=1}^{n}{\left(x_{i}-\bar{x}\right)}^{2}}$$

(9) $$Z=\frac{I-E\left(I\right)}{\sigma \left(I\right)}$$

where I is Moran's I; n is the total number of areas; $x_{i}$ and $x_{j}$ are the changes in CO_2_ emissions or PM_2.5_ concentrations in the ith and jth regions, respectively; $\bar{x}$ is the average change; $W_{ij}$ is the spatial weight matrix; E(I) is the expectation value of I; and σ(I) is the standard deviation of each variable.

Compared with the Global Moran's I, which describes the overall spatial correlation, spatial autocorrelation at the local scale can explain the local correlations and changes between adjacent units [56]. Therefore, to explore the spatial characteristics of CEs, the local indicators of spatial association (LISA) index was used in this study. The formula was shown in following Equation (10):

(10) $$I_{i}=z_{i}\sum_{j}w_{ij}z_{j}$$

where $I_{i}$ is the Local Moran's I; $w_{ij}$ is the spatial weight matrix; and $z_{i}$ and $z_{j}$ are the standardization values of the observed values.

### 2.6 Analysis of the impact factors of synergistic control

As mentioned previously, there could be spatial correlations in CO_2_ emissions or PM_2.5_ concentrations between adjacent cities. Compared to conventional regression models such as the ordinary least squares (OLS) model, spatial econometric models could effectively consider spatial correlations [55]. Thus, to analyze the spatiotemporal heterogeneity of influencing factors, we used the spatial lag model (SLM) and spatial error model (SEM). The SLM was expressed as follows in Equation (11):

(11) Y=ρWY+Xβ+μ

where Y is the vector of a dependent variable; ρ is the spatial lag parameter; W is the spatial weight matrix; X is the vector of explanatory variables; β is the vector of regression coefficients; and μ is the vector of the error term [57,58].

The SEM could be written as follows Equations (12), (13):

(12) Y=α+Xβ+μ

(13) μ=λWμ+ε

where ε is the residual and λ is the spatial autoregressive coefficient that reflects the influences of the residuals of adjacent areas on the residuals of the local area [59].

### 2.7 Data

CO_2_ emission data for 335 cities in China were sourced from the China High Resolution Emission Database (CHRED) 3.0 database [[60], [61], [62], [63]], which was compiled by using bottom-up and enterprise-level gridded emission data. By using public statistical yearbooks, official reports, personal surveys, and field investigations, the China City Greenhouse (CCG) working group [64] verified and cross-evaluated the CHRED 3.0 database and established city-level CO_2_ emission data. City-level PM_2.5_ concentration data for 335 cities were sourced from the China National Environmental Monitoring Centre [65]. Detailed data could be found in Tables S1 and S2.

The GDP and POP data for 2015 and 2020 were sourced from the Statistical Bulletin on the National Economic and Social Development for each city [66] and the statistical yearbook for each province or city [67]. To exclude inflation and other price increases, GDP was converted to a constant price in 2015.

## 3 Results

### 3.1 Performance of synergy controls in China

**Fig. 1:**
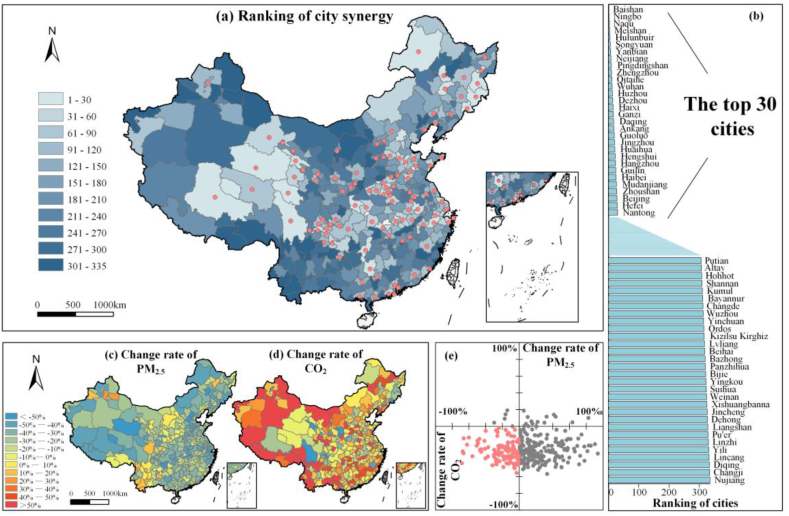
Synergy performance of 335 Chinese cities in the period 2015–2020. (a) Ranking of synergistic effects on CO_2_ emissions and PM_2.5_ concentrations. (b) Top and bottom 30 cities according to the synergistic performance rankings. (c–d) Change rates of (c) PM_2.5_ concentrations and (d) CO_2_ emissions from 2015 to 2020. (e) Quadrant analyses of CO_2_ and PM_2.5_ changes. The pink dots in (a) and (e) represent cities with good synergistic performance (defined as decreases in both CO_2_ and PM_2.5_), and the gray dots represent non-synergistic cities (defined as increases in CO_2_ or/and PM_2.5_). (For interpretation of the references to color in this figure legend, the reader is referred to the Web version of this article.)

From 2015 to 2020, the average concentration of PM_2.5_ in Chinese cities decreased from 46 μg/m^3^ to 32 μg/m^3^, and the average CO_2_ emissions increased from 35.3 million tons to 37.7 million tons. Synergistic reductions in CO_2_ emissions and PM_2.5_ concentrations could be seen at the city level, as shown in Fig. 1 (see Tables S1 and S2 for details). The synergistic performance, represented by the synergy index (ranking) (Fig. 1 (a)), showed a good overall pattern in the northeastern, central, southern, and eastern regions. In particular, Hubei, Zhejiang, and Jilin Provinces, with city-averaged rankings of 76, 80, and 94, respectively (a relatively low value indicates better synergistic performance than a relatively high value), exhibited outstanding performance in terms of the synergistic control of CO_2_ and PM_2.5_. In contrast, the synergistic performance levels were poor in the southwestern, northern, and northwestern regions, as the rankings for Yunnan, Shanxi, and Xinjiang Provinces were 282, 243, and 233, respectively (Fig. 1 (a)).

For intercity comparison, the top 30 cities in the synergy ranking were characterized as either highly urbanized (core cities) or nonindustrial (clean cities). Regional cores and provincial capitals, such as Ningbo, Zhengzhou, Wuhan, Hangzhou, Beijing, and Hefei, were well developed with large populations. Thus, the environmental control measures for CO_2_ and air pollutant emissions at these locations were effective. Other synergistic cities with relatively low levels of energy production and consumption, such as Baishan, Naqu, Ganzi, Guoluo, and Haibei, produced few emissions and were clean. In particular, Baishan, which ranked first among all the cities, released the 13th Five-Year Plan, which is a comprehensive work plan for energy conservation, emission reduction and greenhouse gas emission control in 2017 [68] and accordingly formulated various policies to achieve substantial reductions in CO_2_ and air pollutans. Conversely, most of the 30 cities ranked at the bottom in terms of synergistic performance were considered developing cities, including many autonomous states. These cities had high increase rates of CO_2_ emissions and low removal rates of pollutants due to industrialization and urbanization (Fig. 1 (b)).

Our results showed that city-level differences in synergistic performance mainly depended on CO_2_ emission reductions because city-level PM_2.5_ concentrations were almost decreased, while changes in CO_2_ emissions varied greatly. As shown in Figs. 1(c), 308 out of 335 cities experienced reductions, yet only 27 cities experienced slight increases in PM_2.5_ concentrations between 2015 and 2020, which resulted in obvious decreases in PM_2.5_ concentrations by an annual average of −6.3 % in China. This finding demonstrated the remarkable achievements in terms of air pollution control in the past. However, widespread pollution control measures barely contributed to the overall synergistic performance (Fig. 1 (a) and (c)). In comparison, CO_2_ emissions decreased in 114 out of 335 cities but increased in the remaining 221 cities from 2015 to 2020, leading to a national growth rate of CO_2_ emissions of 2.2 % per year. These variations in CO_2_ emissions primarily contributed to the synergistic performance (Fig. 1 (a) and (d)). For instance, the top 10 cities with high synergy performance were among the top 19 % of cities in terms of CO_2_ emission reduction. The bottom 10 cities with poor synergy performance were among the bottom 9 % of cities in terms of CO_2_ emission reduction (Fig. 1 (b)). Moreover, the great spatial heterogeneity of CO_2_ emission changes was responsible for the synergistic performance distribution. Specifically, the northeastern region exhibited the best CO_2_ emission mitigation and as well the synergistic performance characteristics. CO_2_ control was also obviously conducive to good synergistic performance in midwestern cities (Haibei, Ganzi, and Ankang) (Fig. 1 (a) and (e)). In contrast, the southwestern and northwestern regions featured the poorest synergistic performance. As shown in Fig. 1 (e), numerous non-synergistic cities (199 cities in the lower-right corner), which experienced increases in CO_2_ emissions and decreases in PM_2.5_ concentrations, noted the challenge of balancing economic development and CO_2_ control measures in the future.

### 3.2 Economic feedback to synergy controls

**Fig. 2:**
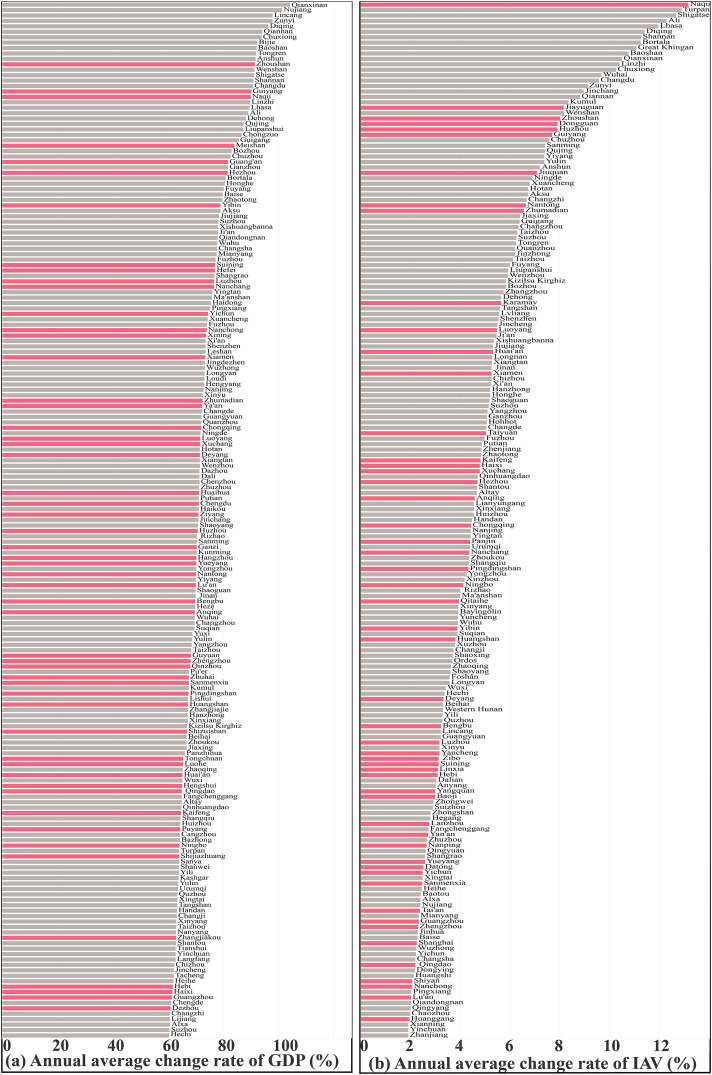
Relationship between economic development and synergistic control for Chinese cities. (a) Cities with GDP change rates above the national average (6 % per year) from 2015 to 2020. (b) Cities with industrial added value change rates above the national average. The pink charts and gray charts are similar to those shown in Fig. 1 (e). (For interpretation of the references to color in this figure legend, the reader is referred to the Web version of this article.)

**Fig. 3:**
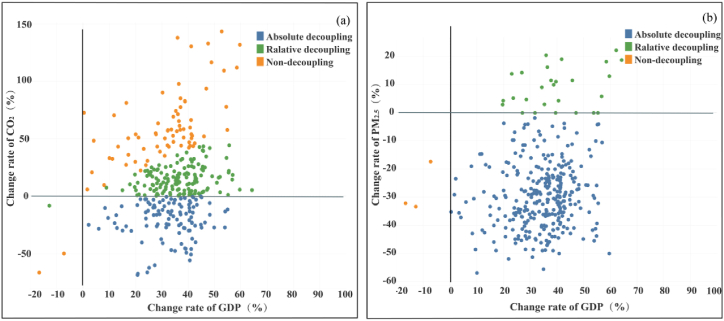
Comparisons of changes in synergy and GDP for Chinese cities. (a) Quadrant analysis of CO_2_ and GDP changes. (b) Quadrant analysis of PM_2.5_ and GDP changes. The colors in (a–b) indicate the decoupling state. (For interpretation of the references to color in this figure legend, the reader is referred to the Web version of this article.)

Fig. 2, Fig. 3 revealed the relationship between economic development and synergistic performance, including rankings of the GDP increase rate and the decoupling status. We observed that economic development and synergistic control characteristics differed among cities. From 2015 to 2020, the average GDP growth rate of each Chinese city was 6.0 % per year, with 191 out of 335 cities exceeding this percentage. Thereafter, only 54 cities performed well in terms of synergistic control, while the remaining 137 cities did not pay sufficient attention to synergistic control during rapid economic development (Fig. 2 (a)). For synergistic cities (54 cities), such as Naqu (GDP change rate: 9.0 % per year, synergy ranking: 3), Meishan (GDP change rate: 8.4 % per year, synergy ranking: 4) and Zhoushan (GDP change rate: 9.2 % per year, synergy ranking: 27), the CO_2_ emissions and PM_2.5_ concentrations were absolutely decoupled from the economy (Fig. 3 (a) and (b)). Similarly, the averaged annual growth rate of industrial added value (IAV) in each city in China was 1.86 %. Among the cities that exceeded the average, only 53 exhibited synergistic control (Fig. 2 (b)). Due to their development of low-carbon economies, these cities simultaneously achieved economic development and synergistic control, providing a framework for similar cities.

However, within the remaining 137 cities, Qianxinan, Nujiang, and Lincang developed fastest but non-synergistically (synergy rankings: 272, 335, and 332) (Fig. 2 (a)). The cities were in the early stages of urbanization and industrialization, consuming energy and emitting CO_2_. Accordingly, economic development was not decoupled from CO_2_ emissions despite somewhat decoupled from pollutants (Fig. 3). These emerging cities should adjust their development mode to bypass carbon-intensive growth and avoid high lock-in effects due to the high difficulty and cost of mature urban retrofitting in the future [25].

**Fig. 4:**
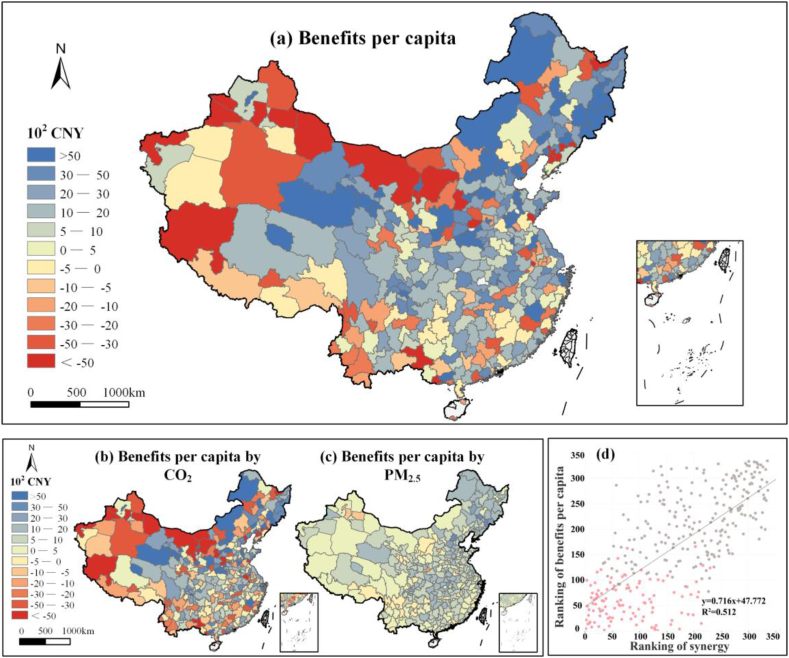
Economic co-benefits from synergistic controls. (a) Benefits per capita from 2015 to 2020 for 335 cities in China. (b–c) Benefits per capita from (b) CO_2_ and (c) PM_2.5_. (d) Correlations between synergy rankings and benefits. The pink dots and gray dots are similar to those shown in Fig. 1 (e). (For interpretation of the references to color in this figure legend, the reader is referred to the Web version of this article.)

Fig. 4 (a) shows the monetized benefits (unit: CNY per capita) of mitigating CO_2_ emissions and PM_2.5_ concentrations from 2015 to 2020 for 335 cities, where values greater than zero represent benefits, otherwise, losses were determined. Our results showed obvious heterogeneity in terms of benefit distributions, which were dominated by the distribution of CO_2_ mitigation benefits. The gross benefits of CO_2_ and PM_2.5_ mitigation were relatively great in the northeastern region (4800 CNY per capita). In particular, Jilin Province received notable a benefit of 6520 CNY per capita (Fig. 4 (a)), and CO_2_ mitigation measured provided a benefit of 4379 CNY per capita, which was two folds to the pollution mitigation benefit of 2141 CNY per capita (Fig. 4 (b) and (c)). This result was associated with the outstanding synergistic controls in multiple cities (e.g., Baishan, Songyuan, and Yanbian) (Fig. 1 (b)). In contrast, the northern and southwestern regions exhibited weak synergy and benefited to a lesser degree than the northeastern region (i.e., they received benefits of 524, and 628 CNY per capita, respectively). Due to significant increases in CO_2_ emissions, the maximum losses increased by −6540 CNY per capita in Xinjiang and −2196 CNY per capita in Inner Mongolia (Fig. 4 (a)). The above economic co-benefits/co-losses were regulated by synergistic performance (R^2^ = 0.51), therefore, major energy-consuming cities should improve their energy structures to mitigate carbon emissions and avoid losses (Fig. 4 (d)).

### 3.3 Mechanisms influencing synergistic control

Different cities have distinct characteristics in terms of economic, social, and environmental aspects. By using spatial correlation analysis, we found that the city-level Global Moran's I was significant for both PM_2.5_ reduction (I = 0.31) and CO_2_ reduction (I = 0.16), with a p value less than 0.01. The local spatial autocorrelation at the city level showed that for PM_2.5_ changes, low-low cities were in the Beijing-Tianjin-Hebei region and the Yangtze River Delta region, while high-high cities were in the northwestern region. For CO_2_ changes, low-low cities were in the central region, while high-high cities were in the western region. In addition, we used SLM and SEM models and selected population, GDP, proportion of secondary industry, proportion of tertiary industry, patents (representing technological innovation), and temperature as impact factors for evaluating the underlying mechanisms of CO_2_ emissions and PM_2.5_ concentrations. As shown in Table S3, the coefficient of GDP was positive for CO_2_ emissions, indicating that economic development increased the quantity of carbon emissions. Additionally, the results demonstrated that the greater the proportion of secondary industry was, the more CEs tended to increase. However, the effects of patents on CO_2_ emissions were significantly negative at the city level, indicating that increasing technological innovation was good for decreasing CO_2_ emissions, possibly because additional patents would enhance technology levels, thereby reducing CO_2_ emission intensities. As shown in Table S4, the coefficients of the proportion of secondary industry and the temperature were significantly correlated with PM_2.5_.

**Fig. 5:**
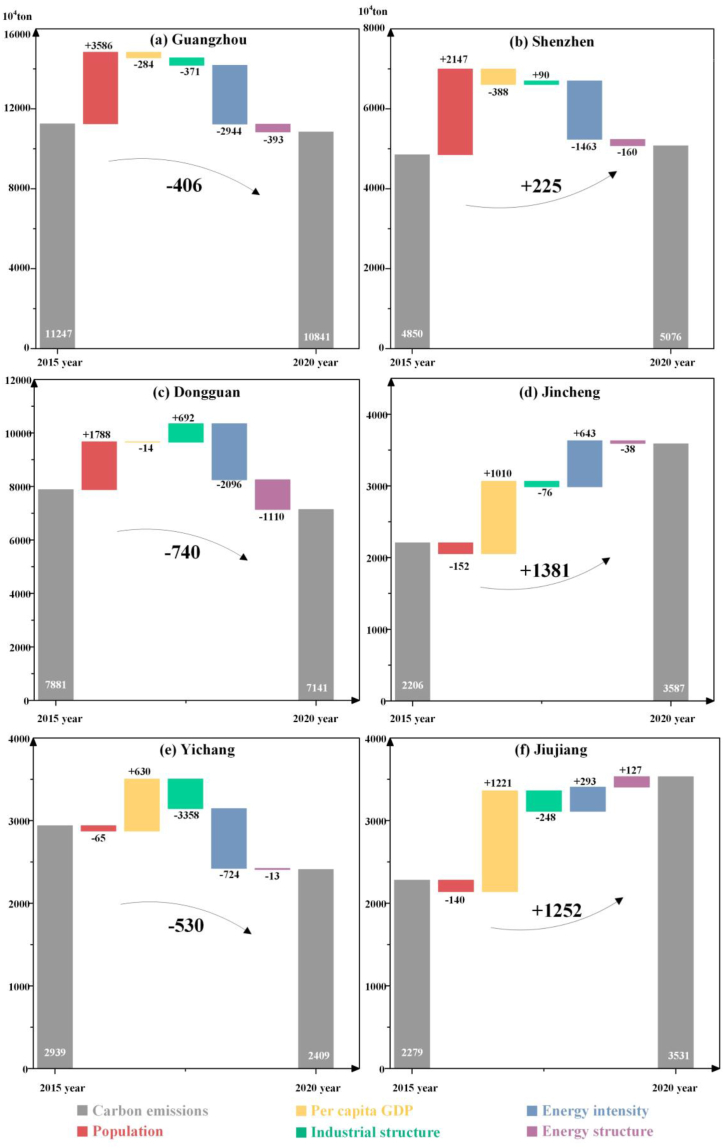
The logarithmic mean Divisia index (LMDI) decomposition analysis in (a–b) service-type cities (Guangzhou and Shenzhen), (c–d) industrial cities (Dongguan and Jincheng), and (e–f) other-type cities (Yichang and Jiujiang).

To determine the mechanisms underlying and factors influencing synergistic control, we analyzed different typical cities, including service-type cities (Guangzhou and Shenzhen), industrial cities (Dongguan and Jincheng), and other cities (Yichang and Jiujiang). For example, Guangzhou and Shenzhen were both fast-growing megacities in the Guangdong-Hong Kong-Macao Greater Bay Area with high levels of economic development and industrialization [69,70]. Although the change rates of GDP and population were similar, the two cities differed significantly in terms of synergistic performance (see Table S1 for details). From 2015 to 2020, Guangzhou achieved synergistic control (the PM_2.5_ concentration and CO_2_ emissions decreased by 36.0 % and 3.6 %, respectively), but Shenzhen did not. CO_2_ emissions in Shenzhen increased from 4.9 to 5.1 E7 tons despite the decrease in PM_2.5_ concentrations from 27 μg/m^3^ to 19 μg/m^3^, which indicated the pivotal role of CO_2_ emissions in determining synergistic performance. For this purpose, we used LMDI to decompose the forces driving CO_2_ emissions, and the results are shown in Fig. 5.

The driving force results showed that energy-related measures, such as reducing energy intensity and adjusting the energy structure, significantly reduced emissions. During the period of 2015–2020, the population was the dominant factor driving carbon emissions growth in both Guangzhou (+3.6 E7 tons) and Shenzhen (+2.1 E7 tons) because the development opportunities and living standards of large cities attracted an increasing number of people to settle there [71,72], resulting in dramatic increases in CO_2_ emissions (Fig. 5 (a) and (b)). In contrast, the decreases in energy intensity were the major contributors to the decreases in CO_2_ emissions in Guangzhou (−2.9 E7 tons) and Shenzhen (−1.5 E7 tons), indicating that the constantly improving energy efficiencies in both cities led to significant reductions in CO_2_ emissions. By comparing the two cities, we found that Guangzhou achieved greater improvements in energy efficiency and structure than Shenzhen, surpassing the emission surge due to population growth and reducing total CO_2_ emissions from 2015 to 2020. In comparison, although the improvements in energy efficiency and structure in Shenzhen substantially reduced CO_2_ emissions, the decline was insufficient to offset all CO_2_ emission growth, requiring further future effort in this Shenzhen field. In addition, for selected industrial cities, LMDI results showed that energy structure adjustment had a pronounced mitigating effect on Dongguan city (−1.1 E7 tons), in contrast, little effect was observed in Jincheng (Fig. 5 (c) and (d)). Energy intensity reduction (as an indicator of energy efficiency improvement) significantly reduced emissions in Dongguan but was a force driving carbon emissions in Jincheng. For other cities, we found that industrial structure improvement and energy-related measures led to significant reductions in Yichang city (−3.3 E7 tons and −7.2 E6 tons) but had less of a mitigating effect (−2.5 E6 tons) or a driving force (+2.9 E6 tons) in Jiujiang city (Fig. 5 (e) and (f)).

From 2015 to 2020, both Guangzhou and Shenzhen took extensive measures to control air pollution and CO_2_ emissions. In addition to taking targeted measures to control air pollution (such as implementing the Ultra-Clean Emissions Work Plan in the electricity sector, developing green transportation, reducing vehicle pollution emissions, eliminating and reducing polluting production capacity, and transforming traditional industries to advanced manufacturing and information technology to optimize the industrial structure) [[73], [74], [75]], Guangzhou attached great importance to mitigating CO_2_ emissions and issuing energy conservation and energy efficiency improvement policies. Specifically, Guangzhou distributed energy conservation targets in all districts, promoted energy conservation and carbon reduction measures in key areas and strengthened the energy control measures of major projects [76]. Guangzhou also proposed a ban on the burning of high-polluting fuels, strictly controlled coal consumption, eliminated bulk coal in industry [76] and encouraged all emission sectors to develop natural gas and renewable energy technologies. Specifically, in the electricity sector, Guangzhou encouraged the shift from coal to natural gas and renewable energy for electricity generation [77] and the comprehensive development of new energy vehicles in the transportation sector [77]. By actively implementing these policies, coal consumption in Guangzhou decreased by 28.6 %, and energy intensity decreased by 25.8 % from 2015 to 2020.

In Shenzhen, various measures have been taken to control CO_2_ and air pollutants [[78], [79], [80]]. These measures largely reduced PM_2.5_ concentrations and controlled CO_2_ emission growth. However, the coal consumption and energy intensity decline rates in Shenzhen were 34.7 % and 6.2 % lower than those in Guangzhou, respectively, limiting their ability to achieve synergistic control.

Based on the synergy mechanisms revealed by Shenzhen and Guangzhou, cities should first control energy consumption and improve energy efficiency. For instance, energy-intensive industries should ensure that their energy efficiency meets international or domestic standards. Low-carbon transport should be encouraged to construct a green public transport system. Second, the development of clean electricity should be emphasized. From 2015 to 2020, the electricity demand increased in Guangzhou and Shenzhen. The total electricity consumption in Guangzhou increased from 7.8 to 10.0 E10 kWh [81], while that in Shenzhen increased from 8.2 to 9.8 E10 kWh [82]. As urban electricity demand is constantly increasing, cities should pay more attention to clean and renewable energy (e.g., wind and solar energy) in electricity generation. Conversely, for imported electricity, cities should focus on the source of electricity generation and promote requirements for the proportion of energy generated that must be green. All these suggestions could help cities enhance their synergistic control in the future.

## 4 Conclusion and policy implications

China is simultaneously facing the challenges of improving air quality and mitigating climate change. Given the urgent need to compare synergistic performance among Chinese cities, we develop a novel index for assessing the synergy between CO_2_ mitigation and air pollution control, and we evaluate the co-benefits of all cities in China during the period of 2015–2020. We further explore the primary cause of differences in synergistic performance by analyzing typical cities. The main findings are as follows: (1) PM_2.5_ and CO_2_ have synergistic effects on economic co-benefits of approximately 4800 CNY per capita, particularly in the northeastern region, however, PM_2.5_ and CO_2_ are poorly synergistic over the northern and southwestern regions, with slight economic benefits of only 524 and 628 CNY per capita, respectively. (2) This spatial distribution of synergistic performance is dominated by CO_2_ emissions, suggesting that cities should primarily focus on improving their energy efficiency and energy structure to mitigate CO_2_ emissions, achieve synergy and avoid losses, as revealed by case studies in typical cities.

This work provides valuable information for Chinese cities in terms of reaching a peak in carbon emissions and formulating air quality policies [83,84]. For national policymakers, the index introduced by this study can be applied widely at different administrative levels (including the national, regional, and city levels) to assess the synergistic performance of important environmental issues (e.g., PM_2.5_ and CO_2_). For managers in each city, rankings of synergistic performance can help them recognize the status of synergy and strengthen intercity and interprovincial efforts to achieve emission mitigation goals. Conversely, as the basic administrative unit in China, our results indicate that cities can achieve synergistic control despite their varying levels of development (economic decoupling status). Therefore, cities at similar development stages can compare their intercity policies of energy and industry, which can lead to useful insights for achieving synergy via optimal pathways.

Nonetheless, this study has several limitations. To avoid complexity, the impacts of the process and other influencing factors, such as the technical choice, abatement cost and benefits, and reduction potential of control measures, are not considered. Future research should be designed to seek the optimized solution: the lowest cost technique for reducing air pollution and CO_2_ emissions. In addition, uncertainty in CO_2_ emissions can be caused by the incompleteness of activity levels and emission factors [85]. Future research should continue to reduce the uncertainty in emissions accounting. Despite these limitations, our results are expected to shed light on the synergistic effects of PM_2.5_ and CO_2_ for cities in China and other countries.

## Data availability statement

Data used in this study can be found in the Supplementary Material.

## CRediT authorship contribution statement

**Li Zhang:** Writing – original draft, Visualization, Formal analysis, Data curation. **Linyi Wei:** Writing – original draft, Formal analysis, Data curation. **Jiaqi Ren:** Visualization, Formal analysis. **Zhe Zhang:** Formal analysis, Data curation. **Ruxing Wan:** Formal analysis, Data curation. **Shuying Zhu:** Writing – original draft, Visualization, Data curation. **Bofeng Cai:** Writing – review & editing, Supervision, Conceptualization. **Jinnan Wang:** Supervision, Conceptualization.

## Declaration of competing interest

The authors declare that they have no known competing financial interests or personal relationships that could have appeared to influence the work reported in this paper.
