# Supplementary material for: City-level synergy and co-benefits of mitigating CO2 emissions and air pollution in China
Source: Heliyon. 2024 Jul 17;10(15):e34667. doi: 10.1016/j.heliyon.2024.e34667 (PMC11336269; doi:10.1016/j.heliyon.2024.e34667)
Supplement: Multimedia component 1 [file mmc1.docx]

**Supplementary Material**

**City-level synergy and co-benefits of mitigating CO_2_ emissions and air pollution in China**

Li Zhang^1, §^, Linyi Wei^1, §^, Jiaqi Ren^2^, Zhe Zhang^3^, Ruxing Wan^4^, Shuying Zhu^3,*^, Bofeng Cai^3,*^, Jinnan Wang^3,*^

1 Ministry of Education Key Laboratory for Earth System Modeling, Department of Earth System Science, Tsinghua University, Beijing 100084, China

2 School of Environment, Beijing Normal University, Beijing 100875, China

3 Center for Carbon Neutrality, Chinese Academy of Environmental Planning, Beijing 100043, China

4 School of Economics and Management, Beijing University of Chemical Technology, Beijing 100029, China

^§^Li Zhang and Linyi Wei contributed equally to this work.

^*^Corresponding authors: Shuying Zhu, Bofeng Cai, and Jinnan Wang

E-mail address: [zhusy@ caep.org.cn](mailto:zhusy@%20caep.org.cn) (S.Z.), [caibf@caep.org.cn](mailto:caibf@caep.org.cn) (B.C.) and [wangjn@caep.org.cn](mailto:wangjn@caep.org.cn) (J.W.)

**This file includes:**

**Supplementary Material Table**

Tables S1 to S4

**Supplementary Material Table**

**Table S1**. Air pollutant concentrations, CO_2_ emissions and synergy ranking for 335 cities in China from 2015 to 2020

| City Name | Area | 2015 | | 2020 | | Synergy Ranking |
| --- | --- | --- | --- | --- | --- | --- |
|  |  | PM_2.5_ (µg/m^3^) | CO_2_ (10^4^ ton) | PM_2.5_ (µg/m^3^) | CO_2_ (10^4^ ton) |  |
| Baishan | Northeast | 50 | 2682 | 28 | 849 | 1 |
| Ningbo | East | 43 | 13111 | 23 | 8930 | 2 |
| Naqu | Southwest | 33 | 33 | 18 | 24 | 3 |
| Meishan | Southwest | 58 | 1412 | 32 | 1030 | 4 |
| Hulunbuir | North | 35 | 8202 | 18 | 6664 | 5 |
| Songyuan | Northeast | 47 | 1764 | 27 | 1279 | 6 |
| Yanbian | Northeast | 36 | 1493 | 21 | 1145 | 7 |
| Neijiang | Southwest | 55 | 3948 | 34 | 2364 | 8 |
| Pingdingshan | Center and South | 84 | 9425 | 51 | 6820 | 9 |
| Zhengzhou | Center and South | 91 | 7426 | 51 | 6310 | 10 |
| Qitaihe | Northeast | 55 | 3428 | 33 | 2537 | 11 |
| Wuhan | Center and South | 67 | 9469 | 37 | 8263 | 12 |
| Huzhou | East | 52 | 3599 | 26 | 3404 | 13 |
| Dezhou | East | 96 | 5612 | 49 | 5283 | 14 |
| Haixi | Northwest | 27 | 2180 | 12 | 2113 | 15 |
| Ganzi | Southwest | 14 | 251 | 9 | 111 | 16 |
| Daqing | Northeast | 44 | 4857 | 28 | 3412 | 17 |
| Ankang | Northwest | 50 | 704 | 32 | 442 | 18 |
| Guoluo | Northwest | 28 | 112 | 16 | 100 | 19 |
| Jingzhou | Center and South | 67 | 1842 | 37 | 1716 | 20 |
| Huaihua | Center and South | 48 | 1034 | 29 | 847 | 21 |
| Hangzhou | East | 54 | 8484 | 30 | 8144 | 22 |
| Hengshui | North | 96 | 1941 | 52 | 1901 | 23 |
| Guilin | Center and South | 47 | 1450 | 29 | 1225 | 24 |
| Haibei | Northwest | 28 | 466 | 19 | 157 | 25 |
| Mudanjiang | Northeast | 47 | 1856 | 31 | 1298 | 26 |
| Zhoushan | East | 28 | 2111 | 17 | 1869 | 27 |
| Beijing | North | 78 | 14220 | 38 | 14864 | 28 |
| Hefei | East | 63 | 5641 | 36 | 5593 | 29 |
| Nantong | East | 55 | 5365 | 34 | 4822 | 30 |
| Huanggang | Center and South | 56 | 2293 | 36 | 1852 | 31 |
| Zibo | East | 86 | 8537 | 54 | 7529 | 32 |
| Zunyi | Southwest | 36 | 3803 | 18 | 4024 | 33 |
| Qingdao | East | 50 | 7950 | 32 | 6700 | 34 |
| Panjin | Northeast | 50 | 5995 | 35 | 1959 | 35 |
| Jiaxing | East | 50 | 4118 | 28 | 4198 | 36 |
| Jiangmen | Center and South | 32 | 3890 | 21 | 2946 | 37 |
| Suining | Southwest | 43 | 585 | 29 | 426 | 38 |
| Zhumadian | Center and South | 71 | 2713 | 45 | 2460 | 39 |
| Ezhou | Center and South | 65 | 2219 | 38 | 2241 | 40 |
| Xiamen | East | 27 | 2323 | 18 | 1731 | 41 |
| Jiaozuo | Center and South | 82 | 4531 | 56 | 3255 | 42 |
| Shaoxing | East | 52 | 4720 | 28 | 5016 | 43 |
| Sanmenxia | Center and South | 69 | 4341 | 48 | 2377 | 44 |
| Hainan | Northwest | 30 | 227 | 19 | 209 | 45 |
| Jining | East | 78 | 10068 | 53 | 7348 | 46 |
| Binzhou | East | 76 | 16276 | 49 | 14239 | 47 |
| Huangshan | East | 33 | 465 | 20 | 459 | 48 |
| Xinxiang | Center and South | 89 | 4669 | 52 | 4774 | 49 |
| Shijiazhuang | North | 85 | 13897 | 58 | 10404 | 50 |
| Yichang | Center and South | 66 | 3339 | 41 | 3253 | 51 |
| Langfang | North | 82 | 3387 | 42 | 3746 | 52 |
| Jiuquan | Northwest | 38 | 1004 | 24 | 974 | 53 |
| Xuchang | Center and South | 78 | 3518 | 53 | 2703 | 54 |
| Huangshi | Center and South | 64 | 3475 | 35 | 3812 | 55 |
| Shiyan | Center and South | 51 | 1256 | 33 | 1147 | 56 |
| Xilingol | North | 16 | 5653 | 9 | 6010 | 57 |
| Jingdezhen | East | 42 | 1783 | 25 | 1827 | 58 |
| Luzhou | Southwest | 55 | 1838 | 38 | 1386 | 59 |
| Guangzhou | Center and South | 36 | 11460 | 23 | 11097 | 60 |
| Great Khingan | Northeast | 23 | 342 | 14 | 346 | 61 |
| Linxia | Northwest | 39 | 752 | 28 | 400 | 62 |
| Suizhou | Center and South | 63 | 513 | 37 | 537 | 63 |
| Lu'an | East | 54 | 1375 | 37 | 1091 | 64 |
| Chengdu | Southwest | 57 | 8966 | 41 | 4855 | 65 |
| Liaocheng | East | 95 | 6142 | 53 | 6768 | 66 |
| Qinzhou | Center and South | 33 | 3236 | 24 | 1761 | 67 |
| Shanghai | East | 50 | 23267 | 32 | 23175 | 68 |
| Anqing | East | 50 | 3467 | 36 | 2434 | 69 |
| Yushu | Northwest | 12 | 221 | 8 | 203 | 70 |
| Tangshan | North | 82 | 27390 | 49 | 28902 | 71 |
| Nanping | East | 26 | 1838 | 19 | 1089 | 72 |
| Kashgar | Northwest | 102 | 1695 | 54 | 1977 | 73 |
| Chongqing | Southwest | 51 | 18592 | 33 | 18511 | 74 |
| Heihe | Northeast | 29 | 918 | 17 | 1018 | 75 |
| Nanchong | Southwest | 56 | 784 | 37 | 761 | 76 |
| Zhuhai | Center and South | 29 | 2081 | 19 | 2051 | 77 |
| Guiyang | Southwest | 33 | 4622 | 23 | 4013 | 78 |
| Jinan | East | 85 | 7270 | 50 | 8075 | 79 |
| Zigong | Southwest | 66 | 648 | 43 | 651 | 80 |
| Foshan | Center and South | 36 | 5508 | 22 | 5856 | 81 |
| Harbin | Northeast | 71 | 6808 | 47 | 6645 | 82 |
| Weihai | East | 36 | 2446 | 26 | 1846 | 83 |
| Enshi | Center and South | 49 | 904 | 27 | 1066 | 84 |
| Baoding | North | 104 | 5226 | 50 | 6404 | 85 |
| Luoyang | Center and South | 68 | 7575 | 51 | 4782 | 86 |
| Zhangzhou | East | 32 | 3778 | 20 | 4017 | 87 |
| Chenzhou | Center and South | 43 | 2072 | 28 | 2100 | 88 |
| Ziyang | Southwest | 39 | 744 | 30 | 367 | 89 |
| Shenyang | Northeast | 71 | 6511 | 42 | 7285 | 90 |
| Huangnan | Northwest | 38 | 41 | 21 | 49 | 91 |
| Jilin | Northeast | 57 | 6164 | 41 | 5157 | 92 |
| Zhangjiajie | Center and South | 49 | 357 | 25 | 445 | 93 |
| Yan'an | Northwest | 41 | 3362 | 32 | 1273 | 94 |
| Meizhou | Center and South | 32 | 2869 | 22 | 2717 | 95 |
| Shuangyashan | Northeast | 42 | 1916 | 26 | 2116 | 96 |
| Tacheng | Northwest | 20 | 2176 | 12 | 2475 | 97 |
| Bengbu | East | 61 | 1630 | 43 | 1511 | 98 |
| Jinchang | Northwest | 30 | 1275 | 20 | 1298 | 99 |
| Huai'an | East | 56 | 3718 | 42 | 2829 | 100 |
| Yancheng | East | 48 | 3672 | 33 | 3587 | 101 |
| Dongying | East | 77 | 3553 | 46 | 4112 | 102 |
| Maoming | Center and South | 30 | 1686 | 21 | 1630 | 103 |
| Baicheng | Northeast | 58 | 1074 | 25 | 1432 | 104 |
| Guyuan | Northwest | 30 | 1246 | 24 | 635 | 105 |
| Luohe | Center and South | 77 | 1018 | 55 | 925 | 106 |
| Fuxin | Northeast | 47 | 2159 | 36 | 1625 | 107 |
| Yibin | Southwest | 53 | 2725 | 40 | 2106 | 108 |
| Laibin | Center and South | 40 | 1357 | 30 | 1091 | 109 |
| Lishui | East | 36 | 871 | 21 | 1039 | 110 |
| Xianning | Center and South | 52 | 1850 | 30 | 2228 | 111 |
| Siping | Northeast | 61 | 2154 | 33 | 2745 | 112 |
| Dazhou | Southwest | 59 | 2355 | 39 | 2464 | 113 |
| Suzhou | East | 55 | 16051 | 33 | 18765 | 114 |
| Tonghua | Northeast | 48 | 1891 | 27 | 2325 | 115 |
| Yongzhou | Center and South | 49 | 1728 | 33 | 1787 | 116 |
| Pingliang | Northwest | 41 | 2031 | 22 | 2660 | 117 |
| Guang'an | Southwest | 43 | 2379 | 32 | 2000 | 118 |
| Dongguan | Center and South | 33 | 7916 | 24 | 7141 | 119 |
| Yueyang | Center and South | 50 | 3544 | 37 | 3080 | 120 |
| Tongliao | North | 51 | 7311 | 34 | 7700 | 121 |
| Fushun | Northeast | 52 | 5841 | 43 | 2935 | 122 |
| Zhangjiakou | North | 30 | 5615 | 23 | 4485 | 123 |
| Ulanqab | North | 38 | 5339 | 22 | 6598 | 124 |
| Tai'an | East | 66 | 6610 | 50 | 5579 | 125 |
| Deyang | Southwest | 48 | 1409 | 37 | 1089 | 126 |
| Tongchuan | Northwest | 52 | 2829 | 43 | 1658 | 127 |
| Huaibei | East | 56 | 8737 | 48 | 2957 | 128 |
| Shantou | Center and South | 30 | 2131 | 19 | 2481 | 129 |
| Hebi | Center and South | 67 | 3233 | 57 | 1697 | 130 |
| Tianjin | North | 68 | 18701 | 48 | 18889 | 131 |
| Xiaogan | Center and South | 68 | 1490 | 35 | 2137 | 132 |
| Karamay | Northwest | 30 | 3766 | 26 | 1508 | 133 |
| Hezhou | Center and South | 37 | 1065 | 27 | 1003 | 134 |
| Wuxi | East | 58 | 7997 | 33 | 10535 | 135 |
| Taizhou | East | 58 | 3269 | 37 | 3809 | 136 |
| Aksu | Northwest | 66 | 2703 | 39 | 3384 | 137 |
| Nanjing | East | 54 | 8636 | 31 | 11458 | 138 |
| Hegang | Northeast | 47 | 1759 | 24 | 2649 | 139 |
| Longnan | Northwest | 35 | 516 | 18 | 776 | 140 |
| Wuhai | North | 47 | 4651 | 32 | 4951 | 141 |
| Quzhou | East | 40 | 3396 | 26 | 3850 | 142 |
| Kaifeng | Center and South | 71 | 2097 | 55 | 1806 | 143 |
| Jinhua | East | 51 | 3448 | 28 | 4980 | 144 |
| Pingxiang | East | 56 | 1599 | 33 | 2048 | 145 |
| Shangqiu | Center and South | 73 | 5028 | 52 | 5124 | 146 |
| Chaozhou | Center and South | 36 | 1597 | 24 | 1778 | 147 |
| Shanwei | Center and South | 26 | 1553 | 18 | 1650 | 148 |
| Shenzhen | Center and South | 27 | 4850 | 19 | 5076 | 149 |
| Turpan | Northwest | 66 | 1419 | 42 | 1702 | 150 |
| Puyang | Center and South | 77 | 1544 | 59 | 1435 | 151 |
| Liupanshui | Southwest | 34 | 5682 | 22 | 6646 | 152 |
| Wenzhou | East | 41 | 3005 | 25 | 3794 | 153 |
| Liaoyang | Northeast | 59 | 3692 | 41 | 3971 | 154 |
| Liuzhou | Center and South | 46 | 3082 | 29 | 3803 | 155 |
| Xiangtan | Center and South | 53 | 3935 | 39 | 3963 | 156 |
| Tongling | East | 55 | 3932 | 35 | 4814 | 157 |
| Xining | Northwest | 37 | 5572 | 35 | 3349 | 158 |
| Xiangyang | Center and South | 72 | 2530 | 52 | 2588 | 159 |
| Datong | North | 35 | 6176 | 31 | 4570 | 160 |
| Tieling | Northeast | 55 | 3529 | 39 | 3741 | 161 |
| Xinyang | Center and South | 67 | 1890 | 40 | 2566 | 162 |
| Lanzhou | Northwest | 42 | 4211 | 34 | 3647 | 163 |
| Changchun | Northeast | 64 | 5688 | 42 | 6667 | 164 |
| Heze | East | 90 | 3369 | 53 | 4800 | 165 |
| Handan | North | 87 | 12050 | 57 | 14242 | 166 |
| Hengyang | Center and South | 51 | 2562 | 35 | 2865 | 167 |
| Dandong | Northeast | 45 | 1504 | 29 | 1819 | 168 |
| Zhenjiang | East | 56 | 5050 | 38 | 5810 | 169 |
| Yulin | Center and South | 37 | 1802 | 28 | 1815 | 170 |
| Jingmen | Center and South | 66 | 2348 | 45 | 2672 | 171 |
| Shizuishan | Northwest | 42 | 6864 | 40 | 4983 | 172 |
| Heyuan | Center and South | 31 | 941 | 22 | 1015 | 173 |
| Wuhu | East | 55 | 4846 | 35 | 6102 | 174 |
| Xingtai | North | 97 | 3661 | 53 | 6281 | 175 |
| Chifeng | North | 38 | 5622 | 25 | 6680 | 176 |
| Xianyang | Northwest | 58 | 3621 | 54 | 2692 | 177 |
| Huludao | Northeast | 52 | 2856 | 42 | 2608 | 178 |
| Cangzhou | North | 68 | 4812 | 47 | 5408 | 179 |
| Yantai | East | 45 | 6254 | 32 | 6803 | 180 |
| Dalian | Northeast | 46 | 6374 | 30 | 7731 | 181 |
| Western Hunan | Center and South | 44 | 670 | 25 | 1060 | 182 |
| Zhongshan | Center and South | 31 | 2118 | 20 | 2652 | 183 |
| Kunming | Southwest | 23 | 4050 | 24 | 2847 | 184 |
| Taizhou | East | 39 | 2625 | 25 | 3392 | 185 |
| Ma'anshan | East | 58 | 4249 | 36 | 6076 | 186 |
| Yichun | East | 38 | 3887 | 31 | 3601 | 187 |
| Hechi | Center and South | 42 | 462 | 25 | 706 | 188 |
| Taiyuan | North | 55 | 8525 | 54 | 6472 | 189 |
| Leshan | Southwest | 51 | 2888 | 35 | 3380 | 190 |
| Yunfu | Center and South | 31 | 1900 | 22 | 2132 | 191 |
| Linyi | East | 74 | 7716 | 48 | 9751 | 192 |
| Weifang | East | 69 | 10151 | 50 | 10981 | 193 |
| Chengde | North | 40 | 4713 | 27 | 5740 | 194 |
| Jiamusi | Northeast | 31 | 1616 | 28 | 1392 | 195 |
| Zaozhuang | East | 84 | 5537 | 55 | 7041 | 196 |
| Jieyang | Center and South | 36 | 1845 | 28 | 1876 | 197 |
| Lianyungang | East | 52 | 3246 | 37 | 3678 | 198 |
| Yangquan | North | 48 | 2748 | 46 | 2309 | 199 |
| Xuancheng | East | 46 | 2554 | 33 | 2876 | 200 |
| Zhangye | Northwest | 35 | 737 | 31 | 667 | 201 |
| Nanchang | East | 41 | 2846 | 33 | 2843 | 202 |
| Suzhou | East | 60 | 1819 | 46 | 1895 | 203 |
| Baoshan | Southwest | 28 | 727 | 19 | 888 | 204 |
| Anshan | Northeast | 70 | 5799 | 44 | 8609 | 205 |
| Baoji | Northwest | 51 | 2765 | 47 | 2413 | 206 |
| Fuzhou | East | 38 | 1255 | 27 | 1447 | 207 |
| Shuozhou | North | 45 | 4198 | 37 | 4093 | 208 |
| Changdu | Southwest | 17 | 69 | 10 | 122 | 209 |
| Shigatse | Southwest | 16 | 33 | 9 | 118 | 210 |
| Zhaoqing | Center and South | 36 | 2511 | 23 | 3621 | 211 |
| Hanzhong | Northwest | 52 | 1493 | 40 | 1568 | 212 |
| Jiayuguan | Northwest | 25 | 3767 | 22 | 3545 | 213 |
| Lijiang | Southwest | 11 | 563 | 12 | 466 | 214 |
| Shangrao | East | 41 | 2571 | 29 | 3039 | 215 |
| Ya'an | Southwest | 32 | 581 | 27 | 576 | 216 |
| Ali | Southwest | 12 | 13 | 7 | 79 | 217 |
| Rizhao | East | 59 | 3775 | 37 | 6034 | 218 |
| Xuzhou | East | 62 | 8998 | 50 | 9265 | 219 |
| Guigang | Center and South | 39 | 2766 | 29 | 3095 | 220 |
| Aba | Southwest | 14 | 240 | 16 | 201 | 221 |
| Loudi | Center and South | 51 | 3752 | 33 | 5485 | 222 |
| Zhoukou | Center and South | 78 | 1115 | 50 | 1686 | 223 |
| Yingtan | East | 39 | 836 | 32 | 852 | 224 |
| Nanyang | Center and South | 69 | 3773 | 51 | 4302 | 225 |
| Jixi | Northeast | 28 | 1556 | 28 | 1432 | 226 |
| Tianshui | Northwest | 36 | 862 | 27 | 976 | 227 |
| Guangyuan | Southwest | 21 | 1189 | 25 | 1021 | 228 |
| Huainan | East | 50 | 5557 | 48 | 5273 | 229 |
| Chongzuo | Center and South | 35 | 1342 | 26 | 1560 | 230 |
| Jinzhong | North | 50 | 5068 | 42 | 5203 | 231 |
| Changzhou | East | 57 | 5965 | 40 | 7532 | 232 |
| Mianyang | Southwest | 41 | 1629 | 34 | 1685 | 233 |
| Dingxi | Northwest | 33 | 692 | 25 | 802 | 234 |
| Ganzhou | East | 38 | 2562 | 26 | 3641 | 235 |
| Yiyang | Center and South | 47 | 1572 | 43 | 1592 | 236 |
| Nanning | Center and South | 38 | 2309 | 26 | 3307 | 237 |
| Haidong | Northwest | 43 | 1383 | 38 | 1417 | 238 |
| Urumqi | Northwest | 60 | 5553 | 47 | 6206 | 239 |
| Shaoyang | Center and South | 54 | 1450 | 38 | 1918 | 240 |
| Yangzhou | East | 52 | 3030 | 36 | 4188 | 241 |
| Liaoyuan | Northeast | 56 | 792 | 39 | 1079 | 242 |
| Qujing | Southwest | 23 | 5272 | 20 | 5457 | 243 |
| Benxi | Northeast | 54 | 3516 | 35 | 6074 | 244 |
| Yuncheng | North | 61 | 7979 | 57 | 8107 | 245 |
| Jinzhou | Northeast | 58 | 1893 | 47 | 2079 | 246 |
| Anshun | Southwest | 24 | 1470 | 23 | 1488 | 247 |
| Quanzhou | East | 26 | 5679 | 21 | 6293 | 248 |
| Suqian | East | 59 | 1778 | 45 | 2126 | 249 |
| Anyang | Center and South | 87 | 4647 | 62 | 6290 | 250 |
| Xinzhou | North | 52 | 3041 | 44 | 3349 | 251 |
| Ji'an | East | 39 | 1606 | 28 | 2242 | 252 |
| Chuzhou | East | 59 | 1559 | 39 | 3377 | 253 |
| Changsha | Center and South | 57 | 2910 | 41 | 4077 | 254 |
| Linfen | North | 54 | 8645 | 52 | 9103 | 255 |
| Longyan | East | 23 | 3509 | 18 | 4172 | 256 |
| Wuzhong | Northwest | 41 | 3267 | 34 | 3703 | 257 |
| Honghe | Southwest | 28 | 3353 | 26 | 3586 | 258 |
| Baise | Center and South | 40 | 2157 | 27 | 4181 | 259 |
| Qingyang | Northwest | 31 | 584 | 28 | 639 | 260 |
| Hotan | Northwest | 78 | 758 | 58 | 987 | 261 |
| Shangluo | Northwest | 38 | 651 | 30 | 775 | 262 |
| Fuyang | East | 49 | 2115 | 49 | 2234 | 263 |
| Bozhou | East | 58 | 843 | 47 | 990 | 264 |
| Huizhou | Center and South | 25 | 3480 | 20 | 4166 | 265 |
| Baiyin | Northwest | 34 | 1918 | 27 | 2311 | 266 |
| Haikou | Center and South | 20 | 516 | 14 | 859 | 267 |
| Fuzhou | East | 27 | 4797 | 21 | 5930 | 268 |
| Hinggan | North | 35 | 1192 | 25 | 1873 | 269 |
| Wuwei | Northwest | 34 | 613 | 35 | 663 | 270 |
| Gannan | Northwest | 27 | 265 | 19 | 480 | 271 |
| Qianxinan | Southwest | 16 | 2329 | 19 | 2456 | 272 |
| Bayingolin | Northwest | 43 | 1419 | 31 | 2185 | 273 |
| Yichun | Northeast | 29 | 844 | 21 | 1299 | 274 |
| Baotou | North | 44 | 10013 | 44 | 11120 | 275 |
| Zhaotong | Southwest | 26 | 1387 | 20 | 1869 | 276 |
| Yangjiang | Center and South | 29 | 1766 | 21 | 2783 | 277 |
| Zhongwei | Northwest | 43 | 1845 | 33 | 2564 | 278 |
| Lhasa | Southwest | 17 | 219 | 12 | 458 | 279 |
| Yuxi | Southwest | 18 | 2618 | 20 | 2885 | 280 |
| Zhuzhou | Center and South | 52 | 1277 | 38 | 1968 | 281 |
| Xinyu | East | 37 | 2249 | 30 | 2819 | 282 |
| Qiannan | Southwest | 19 | 2389 | 17 | 2835 | 283 |
| Zhanjiang | Center and South | 26 | 3909 | 21 | 4972 | 284 |
| Xi'an | Northwest | 53 | 3995 | 51 | 4613 | 285 |
| Qinhuangdao | North | 46 | 3071 | 34 | 4850 | 286 |
| Wenshan | Southwest | 31 | 898 | 23 | 1416 | 287 |
| Dali | Southwest | 21 | 1122 | 16 | 1688 | 288 |
| Sanya | Center and South | 15 | 315 | 11 | 521 | 289 |
| Chuxiong | Southwest | 17 | 730 | 18 | 836 | 290 |
| Chizhou | East | 33 | 2258 | 34 | 2616 | 291 |
| Shaoguan | Center and South | 31 | 2640 | 24 | 3883 | 292 |
| Qiandongnan | Southwest | 27 | 1560 | 24 | 1912 | 293 |
| Bortala | Northwest | 33 | 680 | 24 | 1584 | 294 |
| Changzhi | North | 55 | 4324 | 44 | 6377 | 295 |
| Yulin | Northwest | 33 | 16479 | 33 | 19836 | 296 |
| Fangchenggang | Center and South | 29 | 1534 | 22 | 2725 | 297 |
| Alxa | North | 30 | 1673 | 23 | 2833 | 298 |
| Qingyuan | Center and South | 31 | 3935 | 28 | 4940 | 299 |
| Qiqihar | Northeast | 38 | 2607 | 31 | 3737 | 300 |
| Ningde | East | 28 | 1576 | 22 | 2407 | 301 |
| Chaoyang | Northeast | 41 | 2785 | 35 | 3696 | 302 |
| Jiujiang | East | 48 | 3159 | 38 | 4807 | 303 |
| Sanming | East | 27 | 3379 | 22 | 4867 | 304 |
| Tongren | Southwest | 25 | 1307 | 25 | 1579 | 305 |
| Putian | East | 29 | 843 | 22 | 1944 | 306 |
| Altay | Northwest | 13 | 352 | 10 | 652 | 307 |
| Hohhot | North | 38 | 6127 | 40 | 7476 | 308 |
| Shannan | Southwest | 10 | 89 | 9 | 119 | 309 |
| Kumul | Northwest | 35 | 3440 | 27 | 6297 | 310 |
| Bayannur | North | 40 | 1807 | 33 | 2731 | 311 |
| Changde | Center and South | 48 | 1933 | 41 | 2779 | 312 |
| Wuzhou | Center and South | 33 | 375 | 26 | 715 | 313 |
| Yinchuan | Northwest | 43 | 11140 | 36 | 17450 | 314 |
| Ordos | North | 23 | 15882 | 24 | 20663 | 315 |
| Kizilsu Kirghiz | Northwest | 45 | 255 | 35 | 745 | 316 |
| Lvliang | North | 42 | 2643 | 33 | 8549 | 317 |
| Beihai | Center and South | 27 | 790 | 23 | 1252 | 318 |
| Bazhong | Southwest | 33 | 386 | 28 | 623 | 319 |
| Panzhihua | Southwest | 26 | 1991 | 29 | 2642 | 320 |
| Bijie | Southwest | 25 | 4184 | 24 | 6045 | 321 |
| Yingkou | Northeast | 48 | 3914 | 41 | 6676 | 322 |
| Suihua | Northeast | 36 | 1135 | 41 | 1605 | 323 |
| Weinan | Northwest | 56 | 3703 | 53 | 5737 | 324 |
| Xishuangbanna | Southwest | 26 | 242 | 29 | 347 | 325 |
| Jincheng | North | 51 | 2346 | 46 | 4091 | 326 |
| Dehong | Southwest | 26 | 215 | 22 | 523 | 327 |
| Liangshan | Southwest | 21 | 1377 | 22 | 2251 | 328 |
| Pu'er | Southwest | 20 | 573 | 22 | 1046 | 329 |
| Linzhi | Southwest | 7 | 1 | 7 | 10 | 330 |
| Yili | Northwest | 37 | 2398 | 43 | 4745 | 331 |
| Lincang | Southwest | 23 | 321 | 26 | 743 | 332 |
| Diqing | Southwest | 11 | 112 | 13 | 237 | 333 |
| Changji | Northwest | 44 | 7871 | 53 | 18733 | 334 |
| Nujiang | Southwest | 18 | 64 | 22 | 190 | 335 |

**Table S2**. Statistical data of air pollutant concentrations, CO_2_ emissions and synergy ranking from 2015 to 2020 by region

| Statistical indicators | Area | 2015 | | 2020 | | Synergy Ranking |
| --- | --- | --- | --- | --- | --- | --- |
|  |  | PM_2.5_ (µg/m^3^) | CO_2_ (10^4^ ton) | PM_2.5_ (µg/m^3^) | CO_2_ (10^4^ ton) |  |
| Average | Center and South | 50 | 2790 | 33 | 2875 | 150 |
|  | East | 52 | 4726 | 35 | 5000 | 151 |
|  | North | 54 | 6885 | 39 | 7601 | 184 |
|  | Northeast | 48 | 3048 | 33 | 3138 | 144 |
|  | Northwest | 41 | 2507 | 32 | 2960 | 189 |
|  | Southwest | 30 | 1981 | 24 | 1990 | 205 |
|  | All | 46 | 3529 | 32 | 3770 | 168 |
| Median | Center and South | 48 | 2131 | 29 | 2460 | 147 |
|  | East | 52 | 3553 | 33 | 4112 | 153 |
|  | North | 49 | 5282 | 41 | 6329 | 184 |
|  | Northeast | 48 | 2383 | 33 | 2431 | 131 |
|  | Northwest | 38 | 1493 | 31 | 1584 | 206 |
|  | Southwest | 26 | 1248 | 23 | 1238 | 225 |
|  | All | 44 | 2554 | 32 | 2660 | 168 |
| Variance | Center and South | 350 | 4618668 | 151 | 3713103 | 7889 |
|  | East | 285 | 14962567 | 109 | 14655430 | 7649 |
|  | North | 487 | 29967495 | 156 | 32238321 | 9358 |
|  | Northeast | 147 | 3774591 | 72 | 5042696 | 9243 |
|  | Northwest | 239 | 8783519 | 152 | 18433564 | 9542 |
|  | Southwest | 234 | 8450887 | 87 | 7793868 | 11639 |
|  | All | 350 | 12951077 | 139 | 14907712 | 9380 |

**Table S3**. Results of SLM and SEM for CO_2_

|  | SLM | | SEM | |
| --- | --- | --- | --- | --- |
| Variable | 2015 | 2020 | 2015 | 2020 |
| Constant | -6369.98  (1902.64)*** | -1375.09  (1794.98) | -6572.18  (2091.62)*** | -555.07(2055.81) |
| POP | 1.90(0.78)* | -0.30(0.87) | 2.78(0.81)*** | 0.69(0.93) |
| GDP | 0.98(0.14)*** | 1.09(0.13)*** | 0.88(0.14)*** | 0.99(0.14)*** |
| Sec | 77.26(21.34)*** | 50.70(19.95)** | 98.01(23.16)*** | 60.05(23.16)*** |
| Thd | 77.48(26.50)** | -3.13(25.42) | 78.17(28.43)*** | -5.86(27.31) |
| Tec | -0.12(0.02)*** | -0.07(0.01)*** | -0.10(0.02)*** | -0.07(0.01)*** |
| *ρ* | 0.26(0.06)*** | 0.32(0.06)*** |  |  |
| λ |  |  | 0.39(0.07)*** | 0.44(0.07)*** |
| R2 | 0.57 | 0.57 | 0.59 | 0.59 |
| Log likelihood | -2708.27 | -2726.48 | -2704.99 | -2723.48 |
| AIC | 5430.54 | 5466.96 | 5421.98 | 5458.97 |
| SC | 5456.32 | 5492.75 | 5444.08 | 5481.07 |

**Table S4.** Results of SLM and SEM for PM_2.5_

|  | SLM | | SEM | |
| --- | --- | --- | --- | --- |
| Variable | 2015 | 2020 | 2015 | 2020 |
| Constant | -9.53(7.00) | -3.55(4.04) | 24.43(10.17)** | 18.23(6.51)*** |
| POP | 0.02(0.003)*** | 0.008(0.002)*** | 0.01(0.003)*** | 0.007(0.002)*** |
| GDP | -0.0006(0.0005) | -0.0002(0.0003) | -0.0007(0.0005) | -0.0002(0.0003) |
| Sec | 0.32(0.08)*** | 0.16(0.05)*** | 0.26(0.09)*** | 0.07(0.05) |
| Thd | 0.15(0.10) | 0.11(0.06)*** | -0.02(0.11) | 0.005(0.06) |
| Tec | -4.15e-05(8.31e-05) | -4.92e-05(2.67e-05)* | 5.76e-05(7.99e-05) | -1.61e-05(2.68e-05) |
| Tem | -0.29(0.11)** | -0.15(0.07)* | 0.02(0.33) | 0.11(0.22) |
| *ρ* | 0.74(0.04)*** | 0.75(0.03)*** |  |  |
| λ |  |  | 0.86(0.03)*** | 0.90(0.02)*** |
| R2 | 0.76 | 0.74 | 0.77 | 0.76 |
| Log likelihood | -1076.84 | -948.86 | -1080.88 | -956.24 |
| AIC | 2169.68 | 1913.71 | 2175.77 | 1926.47 |
| SC | 2199.14 | 1943.18 | 2201.55 | 1952.26 |
